# Gut Microbiota Metabolite TMAO and Adolescent Cardiometabolic Health: A Cross-sectional Analysis

**DOI:** 10.1210/jendso/bvaf055

**Published:** 2025-04-05

**Authors:** Fernando de Quadros Iorra, Paula Godinho Rodrigues, Patrícia Martins Bock, Marina Petrasi Guahnon, Sarah Eller, Tiago Franco de Oliveira, Leticia Birk, Patricia de Souza Schwarz, Michele Drehmer, Katia V Bloch, Felipe Vogt Cureau, Beatriz D Schaan

**Affiliations:** Postgraduate Program in Medical Sciences: Endocrinology, Federal University of Rio Grande do Sul, Porto Alegre 90035-903, Brazil; School of Pharmacy, Federal University of Rio Grande do Sul, Porto Alegre 90035-903, Brazil; Post-Graduate Program in Pharmacology and Therapeutics, Federal University of Rio Grande do Sul, Porto Alegre 90035-903, Brazil; Institute of Biological Sciences, Federal University of Rio Grande, Rio Grande 96203-900, Brazil; Postgraduate Program in Epidemiology, School of Medicine, Federal University of Rio Grande do Sul, Porto Alegre 90035-903, Brazil; Pharmacosciences Department, Federal University of Health Sciences of Porto Alegre, Porto Alegre 90050-170, Brazil; Pharmacosciences Department, Federal University of Health Sciences of Porto Alegre, Porto Alegre 90050-170, Brazil; Graduate Program in Health Sciences, Federal University of Health Sciences of Porto Alegre, Porto Alegre 90050-170, Brazil; Graduate Program in Health Sciences, Federal University of Health Sciences of Porto Alegre, Porto Alegre 90050-170, Brazil; Postgraduate Program in Epidemiology, School of Medicine, Federal University of Rio Grande do Sul, Porto Alegre 90035-903, Brazil; Postgraduate Program in Food, Nutrition and Health, School of Medicine, Federal University of Rio Grande do Sul, Porto Alegre 90035-903, Brazil; Institute of Studies in Public Health, Federal University of Rio de Janeiro, Rio de Janeiro 20271-062, Brazil; Graduate Program in Cardiology and Cardiovascular Sciences, School of Medicine, Federal University of Rio Grande do Sul, Porto Alegre 90035-903, Brazil; Postgraduate Program in Medical Sciences: Endocrinology, Federal University of Rio Grande do Sul, Porto Alegre 90035-903, Brazil; Hospital de Clínicas de Porto Alegre, Porto Alegre 90035-903, Brazil

**Keywords:** adolescent, trimethylamine n-oxide, cardiovascular diseases, cardiometabolic health, obesity

## Abstract

**Background:**

Trimethylamine N-oxide (TMAO) is a metabolite derived from gut microbiota that has been associated with cardiovascular and metabolic disease risk in adults. However, its role in assessing cardiometabolic risk in adolescents is unclear.

**Objective:**

This study investigates the association between serum TMAO levels and cardiometabolic health indicators in Brazilian adolescents.

**Materials and Methods:**

This is a multicenter, cross-sectional analysis involving 4446 participants aged 12 to 17 years from four Brazilian cities. Serum TMAO levels were quantified using liquid chromatography-tandem mass spectrometry, and associations with clinical, metabolic, and inflammatory variables were evaluated through multivariate linear regression analyses.

**Results:**

After adjusting for potential confounders, being in the highest tertile of serum TMAO was positively associated with waist circumference [β 1.45; 95% confidence interval (CI) 0.77, 2.14; *P* < .001], body mass index Z-score (β .19; 95% CI 0.10, 0.27; *P* < .001), and C-reactive protein (β .24; 95% CI 0.13, 0.34; *P* < .001). A negative association between the highest tertile of TMAO and fasting plasma glucose was also observed (β −1.22; 95% CI −1.77, −0.66; *P* < .001).

**Conclusion:**

TMAO may serve as an emerging biomarker for cardiometabolic risk assessment in adolescents.

Cardiovascular diseases (CVD), driven largely by ischemic heart disease and stroke, are a leading cause of death worldwide. They also impose a substantial burden of disability-adjusted life-years and healthcare costs [1, 2]. Large population-based cohorts suggest that the traditional and well-established risk factors, such as hypertension, diabetes, dyslipidemia, obesity, and smoking, account for between 50% and 70% of events [3, 4]. In other words, a significant portion of this risk remains unexplained and requires further investigation.

In addition, although the atherosclerotic process typically remains in a preclinical phase until adulthood, it may begin in childhood and adolescence, when risk factors and harmful behaviors become established and start to fuel this pathological process [5-7]. For instance, excess body weight at a young age is linked to a higher risk of CVD and CVD mortality in adulthood [8, 9]. In this scenario, early intervention strategies for weight reduction can alter the course of cardiometabolic conditions, reducing their risk in adult life [10, 11]. Therefore, studies focusing on the cardiometabolic health of the young population, particularly on biomarkers or risk factors for early detection, are essential for the development of risk reduction strategies.

Among emerging CVD risk factors, trimethylamine N-oxide (TMAO) has gained prominence in recent years. TMAO is a gut microbiota-dependent blood metabolite whose formation begins with the ingestion of dietary precursors, such as choline and L-carnitine, which are abundant in some foods, particularly red meat, eggs, dairy products, and fish. Intestinal microorganisms convert these precursors into trimethylamine, which is then absorbed into the bloodstream and converted to TMAO by hepatic flavin-containing monooxygenases (FMO), notably FMO-3 [12, 13]. In addition to dietary sources intake and microbiota composition, TMAO levels are also influenced by individual factors such as age, sex, genetics, and health conditions [13].

Several studies carried out in adult populations have linked this metabolite to an increased risk of CVD, as a biomarker and prognostic factor [14-17]. Moreover, TMAO itself might play a role in the atherogenic process by increasing macrophage foam cell formation, platelet activation, and inducing endothelial dysfunction and inflammation [18]. Finally, correlations have been observed between higher TMAO levels and many other chronic conditions, such as obesity [19], kidney disease [20], diabetes [21, 22], heart failure [23, 24], and increased mortality [16, 17], although these findings are not consistent across all studies [25].

In contrast to the substantial number of studies investigating the relationship between this derivative of the gut microbiota and increased cardiovascular risk in adults, there is a significant lack of studies in the adolescent population, a strategic group in which risk assessment can lead to the establishment of early prevention and intervention strategies. Therefore, we aimed to conduct a multicenter cross-sectional analysis of TMAO serum levels correlating with clinical, metabolic, and inflammatory variables in Brazilian adolescents, looking for a better understanding of the impact of this metabolite on the cardiometabolic health of the youth.

## Materials and Methods

### Study Design, Setting, and Participants

This study is a multicenter cross-sectional analysis of data derived from the Study of Cardiovascular Risks in Adolescents (Portuguese acronym ERICA). A detailed description of the methodology and main results of the original study has been previously reported [26, 27]. In brief, ERICA was a nationwide, school-based study conducted in Brazil between 2013 and 2014 to assess the prevalence of metabolic syndrome and cardiovascular risk factors in adolescents. Teenagers aged 12 to 17 years, attending public and private schools in all 27 federation units, were evaluated through questionnaires and clinical and anthropometric assessments. Those attending morning classes also underwent fasting blood sampling (details are provided in the next section).

Biorepositories were established in 4 capital cities (Porto Alegre, Rio de Janeiro, Brazil, and Fortaleza), where blood samples were stored at −80 °C for future biochemical analysis. As a result, only students with stored blood samples were eligible for inclusion in this study. Additionally, because blood collection for the ERICA study was limited to morning students, only participants from the morning shift were included in the analysis. Those outside the eligible age range at the time of data collection or with missing data for covariates, body mass index (BMI), and blood pressure were also excluded.

The ERICA study followed the principles of the Declaration of Helsinki and was approved by the research ethics committees of all participating centers. Adolescents and their legal guardians provided informed written consent.

### Assessment of Sociodemographic, Behavioral, Anthropometric, and Clinical Variables

Sociodemographic and behavioral aspects were assessed through self-administered questionnaires using a personal digital assistant (LG® GM 750Q, Seoul, Gyeonggi, South Korea). Socioeconomic status was categorized into 3 groups based on the sum of family assets self-reported by the students. Food consumption was assessed through interviews conducted by trained researchers, utilizing a 24-hour food recall. Based on this data, individual consumption of ultra-processed foods was estimated using the NOVA food classification system [28], and the percentage of total energy intake derived from ultra-processed foods was calculated. Medical history including previously known diseases such as hypertension, diabetes, and dyslipidemia was assessed based on self-reported diagnoses.

For anthropometric assessment, weight was determined on an electronic scale (Líder® P200M, Araçatuba, SP, Brazil), height was measured on a calibrated portable stadiometer (Alturexata®, Belo Horizonte, MG, Brazil) and determined by the average of 2 measurements, and waist circumference was evaluated with an inextensible measuring tape, at the midpoint between the lower costal margin of the last rib and the iliac crest in the mid-axillary line [26]. Obesity was classified according to BMI, the ratio of weight (kg) to the square of height (m). The World Health Organization reference O curves were used to classify the weight status of adolescents, with BMI-for-age as the index, adjusted for sex. The adopted cutoff points were Z-score < −3 (severe thinness); Z-score ≥−3 and < −2 (thinness); Z-score ≥ −2 and ≤ 1 (healthy weight); Z-score > 1 and ≤ 2 (overweight); and Z-score > 2 (obesity) [29].

Blood pressure was measured by an automatic oscillometric device (Omron® 705-IT, Bannockburn, IL, USA) validated for adolescents [30], with the subject seated and rested for at least 5 minutes and the appropriately sized cuff positioned on the right arm. Three consecutive measurements were taken, with an interval of at least 3 minutes. For analysis purposes, the average of the last 2 measurements was used.

Blood sampling was obtained only from the students in the morning shift, as fasting for 10 to 12 hours was required. The biochemical tests performed at the time of venipuncture were glycated hemoglobin (HbA1c), fasting plasma glucose, insulin, triglycerides, total cholesterol, low-density lipoprotein cholesterol, and high-density lipoprotein cholesterol fractions. Furthermore, C-reactive protein (CRP), adiponectin, and creatinine were measured posteriorly from the stored samples. The glomerular filtration rate (GFR) was estimated based on creatinine, using the updated Schwartz formula [31]. To assess kidney disease, individuals were classified based on the glomerular filtration rate, with chronic kidney disease (CKD) being considered an estimated GFR < 60 mL/min/ 1.73 m^2^ [32].

### Liquid Chromatography-Tandem Mass Spectrometry for TMAO Analysis

The samples stored in Rio de Janeiro, Brazil, and Fortaleza were all sent to Porto Alegre, preserved overnight in dry ice, and analyzed at the Analytical Center of the Federal University of Health Sciences of Porto Alegre. The TMAO levels were determined in the preserved serum samples by a liquid chromatography coupled triple quadrupole mass spectrometer. A Nexera-i LC-2040C Plus system coupled to an LCMS-8045 triple quadrupole mass spectrometer (Shimadzu®, Kyoto, Kansai, Japan) was used for the analysis. Data acquisition and processing were performed using LabSolutions software (Shimadzu).

### Statistical Analysis

Sample size calculations for the original study (ERICA) were conducted based on the school census at the time of data collection [27]. The present analysis included a relative convenience sample from cities where blood samples were stored [27]. Due to its nonlinear distribution and the lack of a well-established cut-off point in the literature, TMAO was categorized into tertiles for the analyses. The sociodemographic and clinical characteristics of the adolescents were assessed according to the 3 TMAO categories. For the analyses, CRP levels above 10 mg/L were excluded, due to the potential relationship with acute inflammatory states [33]. CRP, insulin, triglycerides, and adiponectin were logarithmically transformed. Cases with missing data for a variable were excluded from the specific analysis but were retained in the overall study sample.

Associations of TMAO levels with cardiometabolic aspects were investigated using linear regression, based on 3 models created to adjust for covariates. The first model included sex, age, skin color, and socioeconomic status. The second model added creatinine, given that TMAO excretion is mostly renal, and the percentage of total energy intake derived from ultra-processed foods, to account for dietary factors. Finally, the third model included, in addition to the variables from the second model, Z-BMI. A sensitivity analysis was performed to evaluate the impact of prior diagnosis or treatment for diabetes on fasting glucose, HbA1c, and insulin levels. Additionally, a comparative analysis of TMAO levels was performed between patients with self-reported comorbidities (hypertension, diabetes, and dyslipidemia) and those without these conditions using the Kruskal-Wallis test.

Statistical analyses were conducted with STATA v. 15 (StataCorp, College Station, TX, USA). All tests were 2-tailed, and a *P*-value less than .05 was considered to be statistically significant. This report follows the Strengthening the Reporting of Observational Studies in Epidemiology statement.

## Results

In the original ERICA study, 72 508 eligible adolescents were identified. Those who underwent the complete assessment including questionnaires, blood pressure, anthropometric measurements, and blood collection totaled 37,504, of which a subgroup from 4 cities had serum samples stored. Finally, after excluding individuals outside the eligible age range or with missing data on covariates, BMI, and blood pressure, 4446 individuals were included in the present analysis (Supplementary Fig. S1 [34]). In the CRP analysis, 683 patients had missing values, and another 156 were excluded due to values above 10 mg/L, enabling the inclusion of 3607 participants. For adiponectin measurement, 517 data points were missing, resulting in a final sample size of 3929. For all other variables, losses were minimal, with none exceeding 25 observations.

The characteristics of the participants are presented in Table 1. The mean age was 14.8 years, they were predominantly female (61.7%) and self-identified as brown skin color (47.7%), and the majority attended public schools (73.7%). Regarding weight status, 69.8% were in the normal range, 18.9% were overweight, and 9.2% were obese. The median of TMAO was 2.40 µmol/L [interquartile range (IQR): 1.60, 4.26], with higher levels in boys (2.66 µmol/L, IQR: 1.73, 4.53) than in girls (2.24 µmol/L, IQR: 1.46, 3.99). In the first, second, and third tertiles, median TMAO levels were 1.33 µmol/L (IQR: 0.93, 1.60), 2.53 µmol/L (IQR: 2.13, 2.93), and 5.46 µmol/L (IQR: 4.26, 7.86), respectively.

**Table 1.** Participant characteristics by TMAO tertiles

	Overall (n = 4446)	First tertile (n = 1530)	Second tertile (n = 1470)	Third tertile (n = 1446)
TMAO, µmol/L, md (IQR)	2.40 (1.60, 4.26)	1.33 (0.93, 1.60)	2.53 (2.13, 2.93)	5.46 (4.26, 7.86)
Age, y, mn (SD)	14.8 (1.5)	14.8 (1.6)	14.8 (1.5)	14.9 (1.5)
Sex, n (%)				
Women	2743 (61.7)	1031 (67.4)	886 (60.3)	826 (57.1)
Skin color, n (%)				
Black	329 (7.4)	99 (30.1)	123 (37.4)	107 (32.5)
Brown	2122 (47.7)	820 (38.6)	688 (32.4)	614 (28.9)
White	1788 (40.2)	526 (29.4)	600 (33.6)	662 (34.7)
Other (yellow, indigenous, not informed)	207 (4.7)	85 (41.1)	59 (28.5)	63 (30.4)
Socioeconomic stratum, n (%)				
First (lowest)	1736 (39.1)	665 (38.3)	573 (33.0)	498 (28.6)
Second	1436 (32.3)	469 (32.7)	473 (32.9)	494 (34.4)
Third	1274 (28.7)	396 (30.0)	424 (33.2)	454 (35.6)
Percent total caloric intake from ultra-processed foods, md (IQR)	28.1 (13.9, 44.8)	28.0 (13.8, 45,0)	28.9 (16.0, 45.4)	30.4 (16.2, 46.1)
BMI category,*^a^* n (%)				
Severe thinness / Thinness	94 (2.1)	33 (35.1)	32 (34.0)	29 (30.0)
Healthy weight	3103 (69.8)	1123 (36.2)	1015 (32.7)	965 (31.1)
Overweight	839 (18.9)	270 (32.2)	276 (32.9)	293 (34.9)
Obesity	410 (9.2)	104 (25.4)	147 (35.9)	159 (38.8)
Cardiometabolic risk factors, mn (SD)				
Waist, cm	72.8 (9.7)	71.9 (8.8)	72.9 (9.8)	73.8 (10.4)
Z-BMI	0.36 (1.18)	0.27 (1.11)	0.35 (1.20)	0.46 (1.22)
Systolic BP, mmHg	110.8 (11.7)	109.8 (11.6)	111.1 (11.7)	111.5 (11.8)
Diastolic BP, mmHg	66.3 (7.8)	66.3 (7.9)	66.2 (7.9)	66.3 (7.9)
Fasting plasma glucose, mg/dL	86.1 (7.8)	86.8 (8.7)	85.8 (7.2)	85.8 (7.5)
HbA1c, %	5.35 (0.35)	5.34 (0.36)	5.34 (0.35)	5.36 (0.36)
Insulin,*^b^* mU/L	8.32 (6.29)	8.40 (6.35)	8.20 (6.25)	8.35 (6.28)
Total cholesterol, mg/dL	151.0 (29.9)	150.3 (29.6)	150.8 (28.0)	151.8 (30.2)
HDL cholesterol, mg/dL	47.9 (11.5)	47.8 (11.5)	48.0 (11.7)	47.9 (11.5)
Triglycerides,*^b^* mg/dL	70.2 (29.7)	69.8 (29.2)	70.0 (29.6)	70.9 (30.3)
Adiponectin,*^b^* ug/mL	11.7 (9.0)	11.6 (9.1)	11.7 (8.8)	11.7 (9.0)
CRP,*^b,c^* mg/L	0.37 (0.48)	0.32 (0.41)	0.38 (0.50)	0.41 (0.53)
Creatinine, mg/dL	0.67 (0.12)	0.65 (0.11)	0.67 (0.12)	0.69 (0.13)
Self-reported diagnosis of previous disease, n (%)				
Hypertension	143 (3.2)	47 (3.1)	45 (3.1)	51 (3.5)
Diabetes	144 (3.2)	49 (3.2)	49 (3.3)	46 (3.2)
Dyslipidemia	407 (9.1)	130 (8.5)	134 (9.1)	143 (9.9)

Data are presented as absolute and relative frequency, mean and SD, or median and IQR, unless otherwise indicated. Among the TMAO tertiles, the percentages for the categories of the variables skin color, socioeconomic stratum, and BMI are calculated by row.

Abbreviations: BP, blood pressure; BMI, body mass index; CRP, C-reactive protein; HbA1c, glycated hemoglobin; HDL, high-density lipoprotein; IQR, interquartile range; md, median; mn, mean; TMAO, trimethylamine N-oxide.

^a^BMI categories according to the World Health Organization classification for Z-BMI for individuals aged 5 to 19 years: severe thinness < −3 SD, thinness: < −2 SD, healthy weight −2 to +1 SD, overweight > + 1 SD, obesity >+2 SD.^29^

^b^Insulin, triglycerides, adiponectin, and CRP were log-transformed and are presented as geometric mean.

^c^CRP levels above 10 mg/L were excluded before logarithmic transformation. Variable with the highest number of losses, final n = 3607.

Table 2 presents the linear regression model adjusted for sex, age, skin color, and socioeconomic status (model 1). According to this model, higher levels of TMAO were positively associated with waist circumference, particularly in the second and third tertiles. For the third tertile, this association was notably stronger. Similarly, a positive association was found between the highest tertile of TMAO and BMI Z-score. Additionally, the second and third tertiles of TMAO were positively associated with the logarithm of CRP.

**Table 2.** Linear regression model adjusted for sex, age, skin color, and socioeconomic status to assess the association of TMAO tertiles with cardiometabolic parameters

TMAO	First tertile (n = 1530)	Second tertile (n = 1470)		Third tertile (n = 1446)	
		β (95% CI)	*P*-value	β (95% CI)	*P*-value
Waist, cm	Ref	0.70 (0.02, 1.38)	**.044**	1.45 (0.77, 2.14)	**<.001**
Z-BMI	Ref	0.08 (−0.01, 0.16)	.067	0.19 (0.10, 0.27)	**<.001**
Systolic BP, mmHg	Ref	0.65 (−0.14, 1.43)	.107	0.77 (−0.03, 1.56)	.059
Diastolic BP, mmHg	Ref	−0.06 (−0.62, 0.50)	.829	−0.09 (−0.65, 0.47)	.755
Fasting plasma glucose, mg/dL	Ref	−1.20 (−1.75, −0.64)	**<.001**	−1.22 (−1.77, −0.66)	**<.001**
HbA1c, %	Ref	0.00 (−0.02, 0.03)	.848	0.02 (−0.01, 0.04)	.207
Insulin,*^a^* mU/L	Ref	−0.01 (−0.06, 0.03)	.503	0.01 (−0.03, 0.05)	.649
Total cholesterol, mg/dL	Ref	0.94 (−1.13, 3.01)	.374	1.96 (−1.13, 4.06)	.067
HDL cholesterol, mg/dL	Ref	0.41 (−0.40, 1.22)	.323	0.43 (−0.39, 1.24)	.306
Triglycerides,*^a^* mg/dL	Ref	0.01 (−0.02, 0.04)	.746	0.01 (−0.02, 0.05)	.370
Adiponectin,*^a^* ug/mL	Ref	0.02 (−0.05, 0.08)	.603	0.01 (−0.06, 0.08)	.762
CRP,*^a,b^* mg/L	Ref	0.18 (0.07, 0.28)	**.001**	0.24 (0.13, 0.34)	**<.001**

Bold *P*-values indicate statistical significance. Abbreviations: BMI, body mass index; BP, blood pressure; CI, confidence interval; CRP, C-reactive protein; HbA1c, glycated hemoglobin; HDL, high-density lipoprotein; TMAO, trimethylamine N-oxide.

^a^Insulin, triglycerides, adiponectin, and CRP were log-transformed.

^b^Levels above 10 mg/L were excluded before logarithmic transformation. Variable with the highest number of losses, final n = 3607.

Still, according to model 1, a negative association with fasting plasma glucose was observed in the second and third tertiles of TMAO. A sensitivity analysis was conducted, excluding cases that reported a previous diagnosis or use of medications for diabetes, due to the potential influence of treatment on glucose levels. However, this did not result in any change in the direction or significance of the observed association. No associations were observed between TMAO and HbA1c or insulinemia, either overall or after excluding those with diabetes. For the other variables, such as systolic and diastolic blood pressure, total cholesterol, high-density lipoprotein cholesterol, triglycerides, and adiponectin, no associations were observed as well (Table 2).

In model 2, the inclusion of creatinine and ultra-processed food consumption as covariates did not substantially influence the associations, and the direction and significance of all of them remained unchanged (Supplementary Table S1 [34]). Finally, the inclusion of the BMI Z-score, tested for variables except for BMI Z-score and waist circumference, did not modify the relationship between TMAO and the other variables, persisting the negative association with fasting plasma glucose and the positive association with CRP (model 3; Supplementary Table S2 [34]).

Furthermore, the comparison of TMAO levels between healthy individuals and those with self-reported hypertension, dyslipidemia, or diabetes revealed no differences (Supplementary Table S3 [34]). However, it is important to note the low prevalence of comorbidities in the studied population. Additionally, only 3 individuals had a GFR within the CKD range, and therefore, no further subgroup analyses were conducted for this group.

## Discussion

The findings of this study indicate a positive association between TMAO levels, BMI Z-score, and waist circumference among adolescents. Notably, there was also a positive association between TMAO and CRP levels, which remained significant even after Z-BMI adjustment, indicating a potential link of TMAO with low-grade systemic inflammation, a well-known mechanism involved in CVD pathogenesis. Furthermore, a negative association between TMAO and fasting plasma glucose levels was identified.

The role of microbiota in the pathogenesis of chronic diseases, particularly CVD and metabolic disorders, has received increasing attention [35-37]. In this context, TMAO is being explored as a potential marker for gut microbiota composition and activity. Although the role of the gut microbiota in TMAO production is well established, the impact of interindividual microbiota variability as a determinant of TMAO levels remains less clear. For example, 1 study showed that TMAO production was higher in young adult men with a higher ratio of Firmicutes to Bacteroidetes [38]. However, another study in healthy subjects failed to identify a clear correlation between microbiota composition and TMAO production, possibly due to limitations in metagenomic assessments that may not fully reflect microbiota functionality [39]. Therefore, even though TMAO levels may offer some insight into microbiota activity, it is important to point out that there are other factors, such as diet, FMO-3 genotype, and kidney function, that influence its levels [40]. Regardless, the pathological correlations observed in humans suggest that, whether as a byproduct of dysbiosis, a disease marker, or even a potential disease promoter, higher TMAO levels may indicate individuals at greater cardiometabolic risk.

### TMAO and Adolescent Health

Given the dearth of research examining the influence of TMAO on adolescent health, this study represents a noteworthy advancement in the field. A search of the MEDLINE database in December 2024 using the keywords “trimethylamine N-oxide” and “adolescents” retrieved 287 articles. Of these, only 17 studies evaluated any aspect of TMAO, such as its generation, metabolomics, or clinical correlations, in a population that included adolescents. Nevertheless, only 4 of the studies in question specifically investigated the relationship between serum TMAO concentrations and cardiometabolic health [41-44].

In a population of children and adolescents, Mihuta et al [41] found results consistent with those observed in our study, particularly with regard to the association between TMAO with BMI and waist circumference. Moreover, they identified significant correlations between TMAO and vascular markers such as carotid intima-media thickness, pulse wave velocity, and blood pressure. Since obesity is 1 of the main risk factors for CVD and other metabolic disorders, the association found between TMAO and weight excess warrants attention. A dose-dependent relationship between TMAO concentrations and increased BMI has been well demonstrated in adults [19]. However, the precise nature of the causal relationship between obesity and TMAO remains to be better elucidated. One hypothesis is that the FMO-3 enzyme, which is a key determinant of TMAO levels, may play a role in the regulation of body adiposity [45]. Additionally, it has been demonstrated that dietary interventions that induce weight loss result in a reduction in urinary TMAO levels in pediatric populations [46, 47]. This provides support for the positive association we observed between TMAO and Z-BMI and offers a potential pathway for the implementation of tailored early intervention strategies.

Furthermore, we observed that TMAO levels were associated with higher CRP, a well-validated inflammatory biomarker. A previous meta-analysis also pointed to a positive association between circulating TMAO and CRP [48]. In vitro models support this finding by demonstrating inflammatory and oxidative stress pathways activated by TMAO [49, 50]. Because elevated CRP levels are useful for predicting atherosclerotic complications in adults and for cardiovascular risk stratification [51-53], the association between TMAO and CRP may indicate a significant link to increased CVD risk in adolescents.

Moreover, there appears to be a synergistic effect between TMAO and inflammation. One study showed that higher TMAO levels prior to percutaneous coronary angioplasty were independently associated with poorer outcomes in patients with acute myocardial infarction complicated by heart failure, particularly in those with elevated CRP levels [54]. Various mechanisms, such as myocardial remodeling, fibrosis, and impaired heart contractility, may contribute to this effect [24]. Review articles have highlighted the association between elevated TMAO levels and the progression of heart failure, suggesting that TMAO may serve as both a marker of disease severity and a potential modulator of cardiovascular remodeling [23, 24].

Conversely, Andraos et al [42] evaluated 1166 children aged 11 to 12 years and did not identify any correlation between TMAO and carotid intima-media thickness, blood pressure or pulse wave velocity, metabolic syndrome score, or high-sensitivity CRP, although associations were observed for TMAO precursors, such as choline, betaine, and carnitine. However, the participants in their study were from a narrower age range than ours and were of Australian nationality, which may introduce differences in genetics, diet, and gut microbiota composition. Previous research has demonstrated that ethnic and geographic factors can influence the relationship between TMAO and clinical outcomes in specific contexts, such as heart failure [55, 56]. This suggests that TMAO may serve as a predictor of outcomes in some populations but not in others.

Additionally, Hsu et al [43] studied 115 children and adolescents with CKD and observed that children with advanced CKD had higher levels of TMAO, but there was no correlation between serum TMAO levels and blood pressure—a finding that we also did not observe in adolescents without CKD. These varying findings reinforce the need for a more comprehensive understanding of TMAO's role in adolescent health and the potential modulating factors that may influence its pathological effects or associations.

An unexpected finding was the negative association between TMAO and fasting plasma glucose, with no associations identified with HbA1c or insulin. Interestingly, Mihuta et al [41] also observed a negative correlation between TMAO and fasting plasma glucose and considered this finding attributable to a greater glycemic variability observed in prediabetic situations [57]. This contrasts with other studies that typically link TMAO to reduced glucose tolerance and higher diabetes risk [21, 22], although conflicting results exist. Some studies report no association [58] or even suggest a protective effect [59] of higher TMAO levels on the incidence of type 2 diabetes. It is also possible that TMAO's effects may differ in younger individuals or vary depending on clinical context or tissue type. For example, studies involving chronic in vivo TMAO administration have shown that it can reduce impaired glucose tolerance in mice fed a high-fat diet [60], while the acute treatment of rat islets with TMAO has been found to reduce β-cell damage and normalize islet function under glycolipotoxic diabetic conditions [61]. Despite these intriguing findings, the precise nature of the relationship between TMAO and glucose metabolism remains unclear and warrants further investigation.

### Strengths and Limitations

The present study has some strengths. First, the inclusion of a large number of individuals from diverse geographical regions within a country such as Brazil, which is characterized by a multiethnic and culturally diverse population, enables a comprehensive representation of the target population. Additionally, the inclusion of predominantly healthy individuals enhances the external validity of the data for adolescents in general. The age range included allows for a more detailed characterization of TMAO in the period that encompasses adolescence, as previous studies have generally included children or only a narrow age range within adolescence. Moreover, we present association data on a wide range of cardiometabolic variables, and the analyses conducted aimed to address confounders based on the adjustment models presented.

On the other hand, it is important to acknowledge some limitations of the study. A single test for TMAO concentration may be susceptible to increased measurement error, including potential interference from factors such as antimicrobial and antifungal use prior to sample collection, for which we have no data. Furthermore, the absence of direct analysis of the fecal microbiota also does not allow us to make inferences regarding the composition of the gut microbiota itself. Additionally, some of the covariates analyzed were self-reported (eg, skin color) or measured on a single visit (eg, blood pressure), which may increase the probability of measurement bias. Finally, the cross-sectional design generates hypotheses but does not allow the determination of causal relationships.

## Conclusion

In conclusion, this study addresses a critical gap in the current research literature by evaluating the impact of TMAO on adolescent health. The identified associations in a predominantly healthy population, particularly with regard to waist circumference, BMI, and CRP, suggest that TMAO may serve as a potential new biomarker for assessing cardiometabolic risk in adolescents. Nevertheless, further elucidation is required. The intricate interplay between dietary habits, gut microbiota, and individual factors in TMAO's metabolism underscores the complexity of its generation and its role as a predictor of outcomes. Prospective studies may help to clarify this relationship and the potential role of TMAO in the early detection of adolescents at increased cardiometabolic risk.

## Data Availability

Some or all datasets generated during and/or analyzed during the current study are not publicly available but are available from the corresponding author on reasonable request.
